# Burnout and Coping Strategies Among Paediatric Oncologists: A Scoping Review

**DOI:** 10.1002/pon.70359

**Published:** 2026-01-05

**Authors:** Harshitha D, Vani Verma, Arun Ghoshal, Divya Sussana Patil, Krithika S. Rao, Seema R. Rao, Amrtavarshini R, Vasudeva Bhat K, Naveen Salins

**Affiliations:** ^1^ Department of Palliative Medicine and Supportive Care Kasturba Medical College Manipal Academy of Higher Education Manipal India; ^2^ Centre for Evidence‐Informed Decision‐Making Prasanna School of Public Health Manipal Academy of Higher Education Manipal India; ^3^ Manipal Hospice and Respite Centre Manipal Academy of Higher Education Manipal India; ^4^ Department of Psychiatry Kasturba Medical College Manipal Academy of Higher Education Manipal India; ^5^ Department of Paediatric Oncology Kasturba Medical College Manipal Academy of Higher Education Manipal India

**Keywords:** burnout, childhood cancer, coping strategies, emotional support, paediatric oncology, psychological distress

## Abstract

**Background:**

Paediatric oncologists routinely face emotionally demanding situations, including prolonged exposure to child suffering, ethically complex decision‐making, and high clinical workloads. These stressors contribute to burnout, moral distress, and psychological strain, yet their lived experiences remain underexplored.

**Objective:**

To synthesise existing evidence on the psychological challenges experienced by paediatric oncologists, with a focus on burnout, emotional exhaustion, moral distress, and coping strategies.

**Methods:**

A scoping review was conducted using the Joanna Briggs Institute methodology and reported in accordance with PRISMA‐ScR guidelines. Five databases [PubMed, ProQuest, Scopus, Web of Science, and CINAHL] were searched for English‐language studies published between 1992 and April 2025. Fourteen studies met the inclusion criteria [5 qualitative, 8 quantitative, 1 mixed‐methods].

**Results:**

Key psychological stressors included emotional exhaustion, depersonalisation, moral distress, and job dissatisfaction. Coping strategies ranged from peer support and physical activity to institutional interventions such as debriefing sessions and resilience training. Stigma surrounding help‐seeking and systemic barriers to emotional support were recurrent themes.

**Conclusion:**

Burnout in paediatric oncology is a multifaceted phenomenon shaped by personal, ethical, and organisational factors. Addressing these challenges requires both individual‐level coping mechanisms and systemic reforms, including trauma‐informed leadership, confidential mental health support, and the redistribution of workloads. Future research should explore longitudinal outcomes and culturally diverse experiences to inform targeted interventions.

## 1 Background

The World Health Organization [WHO] estimates that each year, over two hundred thousand children aged 0–14 years are diagnosed with cancer globally [1]. Moreover, paediatric oncology differs from adult oncology in several key aspects, including the nature of malignancies, treatment protocols tailored to developmental stages, and the significant emotional risk factor involved in treating children, as well as the challenges faced by their parents [2]. Among these challenges, burnout is a notable concern among paediatric oncology professionals [3]. Accordingly, research from the United States indicates that 72% of them experience moderate burnout, while 38% report severe burnout [3], highlighting the impact of job demands and patient safety events on clinician well‐being [4]. Given its implications for paediatric oncologists working in the childhood cancer setting, this scoping review mapped the existing literature, identified key themes, and highlighted areas for future research [5, 6].

Paediatric oncologists address the unique medical and emotional needs of children and their families [7]. Although survival rates in high‐income countries have reached around 80% due to advances in treatment, these improvements have also brought new responsibilities and challenges for healthcare professionals [8]. Along with burnout, several difficulties arise, including moral distress, emotional exhaustion, depersonalization, and reduced personal achievement, which can worsen with high job demands and the emotional impact of patient safety events [4, 8]. Furthermore, the consequences of burnout negatively affect not only healthcare providers but also patient care quality, organisational productivity, and team dynamics [3, 4].

Despite the global significance of cancer and its effects on children, research into the psychological experiences of paediatric oncologists remains limited [3]. Most studies have concentrated on broader aspects of cancer care for children with cancer, leaving notable gaps in understanding burnout and stress among paediatric oncologists [8, 9]. Evidence suggests that institutional interventions, such as counselling, peer support, resilience training, and debriefing sessions, help alleviate distress in paediatric oncology professionals. A positive environment and open communication further reduce burnout and improve patient care [7]. High workloads, long hours, role ambiguity, and a lack of emotional support often exacerbate burnout [4, 10].

In this context, moral distress causes emotional pain resulting from the inability to act by one's ethical beliefs [10]. It frequently arises in acute care settings and in ethically fraught situations, such as transitioning from curative to palliative care [11]. However, a strong correlation exists between burnout and mental health disorders, such as anxiety and depression, among paediatric oncologists [8, 11].

Despite increasing awareness of burnout and emotional distress among paediatric oncologists, significant gaps remain in the literature, particularly in understanding their long‐term psychological well‐being [7, 9]. Notably, they face high emotional demands and prolonged exposure to suffering, placing them at increased risk for burnout and moral distress [3, 7]. A couple of studies have highlighted inadequate staffing as a key factor in paediatric oncology units [8, 9]. However, gaps persist due to methodological limitations and the lack of longitudinal studies [8]. Addressing these issues is crucial for supporting healthcare workers and enhancing patient care [8]. Previous research lacks depth in recognizing coping strategies, creating a significant gap in understanding how to effectively address their psychological well‐being [10]. Addressing these gaps is essential to support healthcare professionals, enhance care delivery, and sustain the resilience of paediatric oncology teams. [9], needful to conduct this study.

Paediatric oncologists work in an emotionally demanding environment that places them at risk of burnout, emotional exhaustion, and moral distress. Although these challenges are widely recognised, existing evidence is fragmented across diverse study designs and settings. This scoping review therefore synthesises current evidence on the psychological challenges faced by paediatric oncologists, focussing on burnout, emotional exhaustion, moral distress, and coping strategies.

## 2 Methods

We conducted this scoping review following the Joanna Briggs Institute [JBI] methodology [5]. The protocol was registered with the Open Science Framework [OSF] at https://doi.org/10.17605/OSF.IO/DC6WJ.

### 2.1 Review Question

What is the existing evidence on the psychological experiences of paediatric oncologists, including burnout, work‐related stressors, and coping strategies?

### 2.2 Eligibility Criteria

#### 2.2.1 Participants

Studies that specifically focussed on paediatric oncologists or reported disaggregated data for them within mixed oncology groups were included. Studies focussed exclusively on adult oncologists or other healthcare professionals [e.g., nurses, social workers, psycho‐oncologists] were excluded.

#### 2.2.2 Concepts

Emotional and psychological experiences such as burnout, emotional exhaustion, moral distress, coping strategies, resilience, and job satisfaction were central to our search and synthesis.

#### 2.2.3 Context

Only studies conducted in paediatric oncology or childhood cancer units were included.

#### 2.2.4 Study Design

Empirical studies [qualitative, quantitative, or mixed‐methods] published in peer‐reviewed journals were eligible. Editorials, commentaries, letters, grey literature, and book chapters were excluded.

### 2.3 Information Sources and Search Strategy

We searched the following databases: PubMed [Medline], ProQuest, Scopus [Elsevier], Web of Science, and CINAHL. Additional studies were identified using Google Scholar and through manual reference checks. The search was limited to English‐language publications between 1992 and April 30, 2025. Boolean operators [AND, OR] were used to combine keywords such as ‘burnout,’ ‘coping strategies,’ ‘paediatric oncologist,’ and ‘emotional distress.’ [Please see Supporting Information S1 for details].

The search was conducted in three stages:

1. Initial limited search to identify relevant keywords and index terms;
2. Comprehensive search using all identified terms;
3. Screening of reference lists from included articles.

### 2.4 Selection Process

**FIGURE 1:**
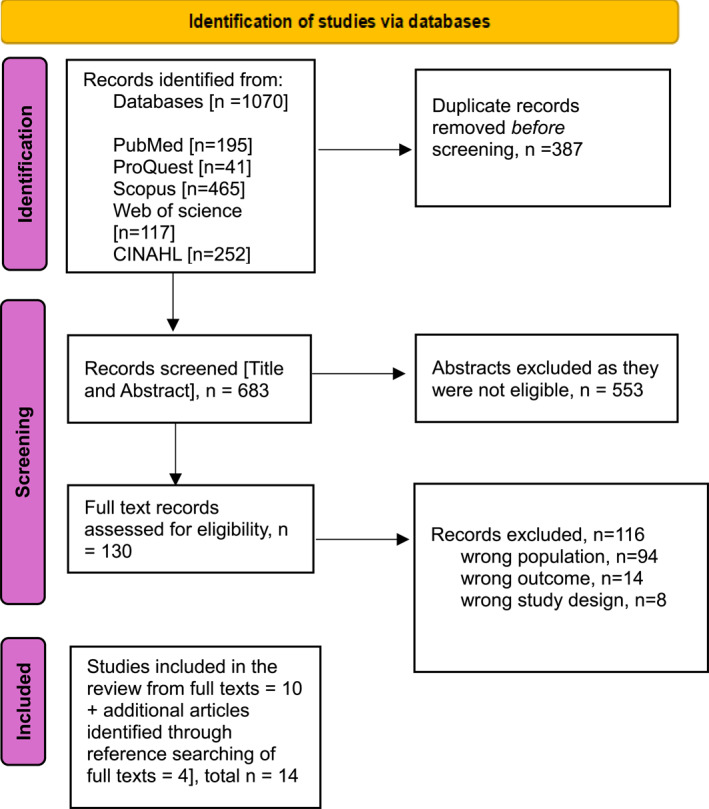
PRISMA ScR flow chart.

All citations were imported into Rayyan for reference management. Duplicates were removed. Two reviewers independently screened titles and abstracts against predefined inclusion criteria. Full texts of potentially relevant articles were retrieved and assessed. Disagreements were resolved by discussion with a third reviewer. The selection process is summarized in a PRISMA‐ScR flow diagram [please see Figure 1 and Supporting Information S2: PRISMA‐ScR Checklist] [6].

### 2.5 Data Synthesis

Data synthesis followed the JBI Manual for Evidence Synthesis for scoping reviews [5]. Given the diverse study designs and outcomes, a narrative synthesis approach was used to descriptively summarise findings across key domains. To guide this process, we applied Popay et al.’s narrative synthesis framework, which supports integration of heterogeneous qualitative and quantitative evidence and was appropriate given the variability across included studies that prevented quantitative pooling [12].

### 2.6 Statistical Methods

Given the scoping review design, this study did not involve primary statistical analysis. However, we systematically extracted and synthesised statistical data reported in the included quantitative and mixed‐methods studies.

## 3 Results

The literature search initially identified 683 articles after removing duplicates. Of these, 130 studies were reviewed in full text. After the eligibility assessment, 10 studies were included in the review. An additional four articles were found through reference searches of the included studies, bringing the total to 14 studies: five qualitative, eight quantitative, and one mixed‐methods study. These studies primarily employed cross‐sectional survey designs and utilized validated instruments, such as the Maslach Burnout Inventory [MBI], to assess burnout dimensions—emotional exhaustion, depersonalization, and reduced personal accomplishment.

Due to methodological heterogeneity across studies—including variations in sample size, measurement tools, and outcome definitions—a meta‐analysis was not conducted. Instead, a narrative synthesis approach was adopted to map key findings and identify patterns across studies. This synthesis was guided by the thematic domains of burnout, moral distress, coping strategies, and organisational factors.

The characteristics of the included studies are summarised in Table 1. Thematic analysis revealed four key domains relevant to the psychological challenges faced by paediatric oncologists.

**TABLE 1:** Summary of included studies: Design, context, and key findings.

Sl No	Title of study, author, year, and place of origin	Type of study	Aims	Study population	Key findings	Gaps identified
1	A critical ethnographic evaluation of paediatric haematology/oncology physicians and burn‐out, Paula Mahon, 2019, Canada	Qualitative study using critical ethnography	To ascertain what affects paediatric haematology/oncology physicians' professional and personal attitudes within their work environment	11 paediatric oncologists from a unit in western Canada	Key themes included responsibility and burnout, shifting of power and educational roles, strong relationships with families and patients, and frustration with bureaucratic systems. Physicians valued long‐term relationships, but these also contributed to burnout. Suggestions included sabbaticals and reducing bureaucracy.	Conducted in a single setting; excluded views of administrators, families, and other staff; not generalizable. Future research should explore administrator perspectives and multicentric experiences.
2	Associations of job demands and patient safety event involvement on burnout among a multidisciplinary group of paediatric haematology‐oncology clinicians, Dunn TJ et al., 2021, USA	Quantitative cross‐sectional survey study	To examine the relationships between job demands, involvement in patient safety events [PSEs], and burnout in paediatric haematology‐oncology clinicians	281 clinicians [including paediatric oncologists, nurse practitioners, nurses, and others] in paediatric haematology‐oncology settings	High job demands and involvement in PSEs were significantly associated with increased emotional exhaustion and depersonalization. Positive team dynamics and supervisor support were protective against burnout.	Cross‐sectional design limits causal inference. Response rate and self‐reported data may introduce bias. Longitudinal studies are needed to examine cause–and–effect relationships.
3	Burnout and perceptions of stigma and help‐seeking behaviour among paediatric fellows; Anna K. Weiss, Sheila M. Quinn, Amy L. Danley, Kandi J. Wiens, Jay J. Mehta [USA, 2021]	Cross‐sectional survey study	[1] Measure burnout among paediatric fellows, [2] assess perceptions of stigma about help‐seeking, [3] explore the relationship between burnout and willingness to seek behavioural health counselling	288 paediatric subspeciality fellows at a freestanding children's hospital [152 responded]	53% met the threshold for burnout. Fellows with burnout perceived more stigma towards mental health help‐seeking. 68% believed that seeking help would affect their future employment. Many would hide their mental health treatment history.	Single‐centre study limits generalizability. Small samples in some departments. No data on race/ethnicity. Nonresponse bias is possible.
4	Burnout, staff support, and coping in paediatric OncologyLiakopoulou M, Panaretaki I, Papadakis V, et al., 2008, Greece	Quantitative cross‐sectional survey	To investigate burnout and coping strategies among paediatric oncology staff and examine the role of social support	42 paediatric oncology staff members [paediatric oncologist, nurses, psychologists] in a Greek tertiary hospital	Emotional exhaustion and depersonalization were prevalent; staff receiving more social support [especially from colleagues] reported lower burnout. Active coping strategies were more prevalent than avoidance strategies.	Small sample size and single‐site limit generalizability. No follow‐up to assess long‐term outcomes. Did not analyse discipline‐specific trends in depth.
5	Demands and rewards associated with working in paediatric oncology, Dix D, Gulati S, Robinson P, Syed I, Klassen A, 2012, Canada	Qualitative	To explore the work‐related demands and rewards experienced by paediatric oncology healthcare providers	33 healthcare providers [10 oncologists, 3 residents, 9 nurses, 5 social workers, 6 child life specialists] from 4 canadian paediatric oncology centres	Identified five core domains: Working with children, families, teams, units, and systems. Work was meaningful but emotionally taxing. Role conflict, workload, and palliative transitions were key stressors.	Need for more research on coping strategies and burnout prevention. The study is limited by a qualitative design and a single‐country context.
6	Exploring moral distress in paediatric oncology: A sample of registered practitioners, Kate Pye, 2013, United Kingdom	Qualitative study using hermeneutic phenomenology	To explore the perceptions of doctors and nurses in a paediatric oncology unit regarding their experiences of moral distress and its impact on team dynamics	8 participants: 4 nurses and 4 paediatric oncologists from a paediatric oncology unit in an English hospital	Moral distress arises from emotional burdens, lack of voice in decision‐making, poor communication, and complex ethical dilemmas. Key themes include decision‐making processes, conflict over treatment decisions, and team communication. Nurses often feel powerless, while doctors sometimes avoid confrontation. Experience influences coping ability.	Limited exploration of how moral distress evolves across experience levels and specialities; calls for future research on how experience influences coping mechanisms; need for better education and support systems
7	*Job Stress and Satisfaction Among the Staff Members at a Cancer Centre*, Peteet JR, Murray‐Ross D, Medeiros C, Walsh‐Burke K, Rieker P, Finkelstein D, 1989, USA	Quantitative survey study	To assess job stress and satisfaction among interdisciplinary staff at a cancer centre and understand the influencing factors	130 staff members, including paediatric oncologists, nurses, social workers, psychologists, chaplains, and administrators at a U.S. cancer centre	Staff reported a high emotional investment in patient care, finding it both rewarding and a major stressor. Satisfaction was highest in roles with autonomy and collegiality. Stress was linked to systemic constraints and lack of emotional support, particularly in end‐of‐life care.	Study lacked longitudinal follow‐up; generalizability limited to one institution; did not explore specific coping mechanisms in‐depth
8	Moral distress and ethical climate in paediatric oncology care impact healthcare professionals' intentions to leave, Ventovaara P, Af Sandeberg M, Blomgren K, Pergert P, 2023, Sweden	Quantitative cross‐sectional survey	To examine the relationships between moral distress, ethical climate, and the intention to leave among healthcare professionals in paediatric oncology	226 healthcare professionals [nurses, paediatric oncologists, and others] from paediatric oncology units across Sweden	Higher levels of moral distress and negative perceptions of ethical climate were significantly associated with a higher intention to leave the profession. Nurses reported higher moral distress than physicians.	The cross‐sectional design limits causal conclusions. Further research is needed to improve the ethical climate and reduce distress among various professional groups.
9	Prevalence of burnout syndrome in health professionals of an onco‐haematological paediatric hospital, Zanatta & Lucca, 2015, Brazil	Exploratory descriptive cross‐sectional study	To identify the prevalence of burnout syndrome among different health professionals	188 health professionals [paediatric oncologist, nurses, nursing technicians] from a paediatric onc‐haematology hospital	High levels of depersonalization [29.8% among nurses] and low personal accomplishment [27.8% among doctors] were common across all groups, as was emotional exhaustion. 4.8% had all three burnout domains.	Small sample for physicians and nurses; limited generalizability; doesn't explore protective or systemic interventions
10	When a child dies: Paediatric oncologists' follow‐up practices with families after the death of their child, Leeat Granek et al., 2015, Canada & Israel	Qualitative study using grounded theory	To examine the follow‐up practices of paediatric oncologists with bereaved families after a child's death	21 paediatric oncologists from two canadian hospitals [ontario]	Paediatric oncologists engage in follow‐up practices, including sending condolence cards/emails, making phone calls, attending funerals/visitations, meeting with families, and participating in memorials. Practices vary due to emotional, logistical, and personal reasons. Long‐term relationships with families were common.	Lack of standardized bereavement protocols, emotional difficulty, and logistical constraints hinder consistent follow‐up. Need for institutional support and medical education in bereavement care.
11	Never enough time: Mixed methods study identifies drivers of temporal demand that contribute to burnout among physicians who care for paediatric haematology‐oncology patient. Lindsay J. Blazin et al.2021, st. Jude Children's research Hospital, Memphis, Tennessee, USA	Mixed methods [quantitative survey + qualitative interviews]	To identify the prevalence and drivers of burnout among physicians caring for paediatric haematology‐oncology [PHO] patients	53 paediatric oncologists [PHO and non‐PHO]; 26 interviews with PHO paediatric oncologists	28% of physicians met criteria for burnout; the main contributing factor was temporal demand. Key drivers: Frequent meetings, insufficient support staff, work‐life imbalance, and administrative overload.	Study limited to a single academic centre; results may not be generalizable. Interventions suggested but not yet tested for efficacy. Broader demographic and multi‐institutional data needed.
12	Oncologists' protocol and coping strategies in dealing with patient loss Leeat Granek, Paolo Mazzotta, Richard Tozer, Monika K. Krzyzanowska 2013, Ontario, Canada	Qualitative study using grounded theory	To explore what protocols [if any] exist for dealing with patient death and what coping strategies oncologists use	20 oncologists from three oncology centres in Ontario [varied by size and career stage]	There are no formal institutional protocols for patient loss; oncologists use ad hoc responses, such as condolence cards, phone calls, and attending rituals. Coping strategies included social support [mainly spouse or nurses], hobbies, exercise, religion, compartmentalization, and emotional withdrawal. Talking to colleagues was rare due to fear of judgement.	Lack of standardized bereavement protocols; emotional support within the professional community is limited due to stigma. Need for institutional interventions and protected time for bereavement practices.
13	Stress‐resilience capacity of paediatric oncologists: A Swedish nationwide and population‐based study of motivation, emotional distress, and overall life satisfaction, Margaretha Stenmarker, Kerstin Palmérus, Ildikó Márky, Paediatric blood & cancer, 2009 [online Dec 2008], Sweden	Cross‐sectional nationwide survey	1. Describe the background characteristics, motivational factors, and coping resources of paediatric oncologists 2. Analyse differences by experience and patient volume. 3. Identify factors influencing stress‐resilience capacity.	89 Swedish paediatric oncologists [out of 105 contacted; 88% response rate]. Included both current and retired professionals across academic and non‐academic centres.	High motivation driven by the need for advanced knowledge, teamwork, and structured routines. High levels of resilience, low emotional distress, and good life satisfaction were reported. More experience is linked with stronger motivation and lower somatisation. Future life satisfaction and low depression levels were strong predictors of stress‐resilience. Lack of time, not patient suffering, was seen as the most stressful burden.	No control group. Cross‐sectional design limits causal interpretation. Possible social desirability bias Cultural specificity may limit generalizability. Personality and psychological factors to be explored in future studies.
14	Career Burnout Among Paediatric Oncologists Michael Roth, Kerry Morrone, Karen Moody, Mimi Kim, Dan Wang, Alyson Moadel, Adam Levy, 2011, USA [montefiore, New York]	Cross‐sectional international survey using the maslach burnout inventory [MBI]	To assess the prevalence of burnout among paediatric oncologists and identify related risk and protective factors	410 paediatric oncologists from the US, Canada, and 11 other countries [response rate: 40%]	38% had high levels, and 72% had moderate to high levels of burnout.Burnout was more common in women and those with < 10 years' experience. Lack of control over work schedule and life dissatisfaction were strong predictors of burnout. Institutions with debriefing forums or support services had lower burnout rates. Only 40% had access to support services.	Possible responder bias and underrepresentation of private/non‐academic centres. Causal relationships cannot be determined from cross‐sectional data Limited generalizability due to a 40% response rate and email‐based recruitment Usage of services was not measured—only their availability was.

### 3.1 Psychological Challenges of Paediatric Oncologists

Paediatric oncologists face a range of psychological challenges arising from the emotional intensity of their work, with studies consistently reporting high levels of burnout and emotional exhaustion [1, 2]. Frequent exposure to patient suffering, complex treatment decisions, and end‐of‐life care contributes to moral distress and significant emotional burden [3]. These challenges occur across diverse clinical and organisational settings, shaped by workload demands, communication difficulties, and systemic constraints that influence clinicians' psychological well‐being [1, 2].

### 3.2 Burnout: Emotional and Physical Exhaustion

Burnout, characterised by chronic workplace stress, emotional exhaustion, depersonalisation, and a diminished sense of personal achievement, was a predominant finding across studies [3, 8]. High levels of burnout were reported among paediatric oncology professionals, with emotional exhaustion and reduced personal accomplishment being the most pronounced dimensions [13].

Several studies highlighted that long‐term therapeutic relationships with patients and their families were emotionally taxing for paediatric oncologists [14]. Beyond the emotional impact of patient suffering, core stressors included excessive workload and insufficient time during clinical hours [15]. One clinician expressed, *‘There's this huge silence after a patient dies… it feels like we're expected to just move on,’* reflecting the emotional toll and a lack of institutional mechanisms for processing grief [14].

This persistent emotional strain often leads to difficulties in detaching from work, with stress infiltrating personal lives and straining relationships [2]. Oncologists described the burden of managing clinical, academic, and administrative duties, frequently with inadequate support staff [15]. Grief over child deaths and the emotional challenge of delivering bad news further compounded their psychological strain, often resulting in feelings of helplessness and diminished well‐being [2, 14].

In response, many clinicians developed emotional detachment as a coping mechanism. While this depersonalisation might temporarily shield them from grief, it also contributed to professional disengagement, health issues, and guilt over patient losses [8]. Communication difficulties and conflicting perspectives on treatment goals further intensified emotional distress [16].

### 3.3 Stressors: Psychological Distress and Moral Strain

Beyond physical exhaustion, paediatric oncologists also experience significant psychological and moral strain. These stem from a deep sense of responsibility for treatment outcomes, relapses, and patient deaths [14]. High patient volumes, resource limitations, and the emotional toll of end‐of‐life care further contribute to distress [1, 7].

A central feature of this strain is moral distress, the emotional pain that arises from being unable to act in accordance with one's ethical beliefs due to systemic or institutional constraints. One oncologist reflected, *‘Sometimes I feel that I'm compromising my own moral values because of what the system demands from me,’* highlighting the internal conflict that arises in ethically complex care scenarios [10].

This distress is particularly prominent during transitions from curative to palliative care, where clinicians may feel unsupported by institutional structures and struggle with decision‐making [3]. Limited organisational support during adverse events and emotionally challenging interactions with patients and families intensifies these moral burdens. To cope, clinicians reported using personal strategies such as spending time with loved ones, maintaining routines, and engaging in physical activity [2].

### 3.4 Stressors: Organisational and Job‐Related Challenges

Systemic issues within paediatric oncology settings often exacerbate burnout and dissatisfaction. Negative experiences during training, time pressures, and high administrative burdens were commonly reported as factors that erode job satisfaction [11, 15]. One clinician shared, *‘The workload just keeps increasing, and there's never enough time to do what's actually important,’* reflecting frustration with chronic time constraints [15].

Additional contributors to job dissatisfaction included institutional culture, lack of autonomy, and procedural bureaucracy—sometimes described as “institutional bullying” which hindered effective care delivery [2]. Clinicians also expressed concern over poor communication with grieving families, who often felt that end‐of‐life discussions lacked empathy and clarity [14].

Alarmingly, many paediatric oncology fellows reported a reluctance to seek psychological help. In one study, 75% of respondents admitted they would conceal their use of mental health services for fear of professional repercussions [11]. This stigma perpetuates emotional isolation and reduces the effectiveness of available support systems.

### 3.5 Coping and Resilience Strategies

Despite these challenges, paediatric oncologists actively develop coping strategies to maintain their emotional well‐being. Common approaches included engaging in regular physical activity, maintaining boundaries between work and personal life, and spending quality time with family [9]. Informal peer support networks and multidisciplinary collaboration also provided a buffer against burnout.

Supportive work environments that encourage open communication, debriefing sessions, and leadership advocacy were found to reduce emotional strain and foster resilience [9]. One oncologist noted, *‘You just develop this armour over time… It's how you survive in this field,’* illustrating the gradual psychological adaptation required to endure the rigours of paediatric oncology [14].

Although stigma surrounding mental health persists, especially among trainees, the presence of confidential counselling services, well‐being programs, and protected time for emotional recovery can significantly reduce barriers to accessing support [2, 11, 17]. Shared emotional processing within teams and institutional encouragement of psychological self‐care further enhanced resilience and professional fulfilment [9].

Despite providing valuable insights, the existing literature on the psychological challenges faced by paediatric oncologists remains limited. Most studies originate from high‐income countries, with minimal representation from low‐ and middle‐income settings, where resource constraints are prevalent [18, 19] Additionally, the field lacks standardised instruments to assess moral distress, emotional burden, and coping, leading to inconsistent measurement across studies. Very few studies adopt longitudinal or intervention designs, making it difficult to understand changes over time or the effectiveness of support strategies [20]. Evidence on institutional responses, team‐based interventions, and culturally relevant wellness frameworks is also scarce, highlighting the need for more robust, diverse, and methodologically rigorous research [20].

## 4 Discussion

This scoping review consolidates existing knowledge on the psychological challenges experienced by paediatric oncologists and applies Maslach and Leiter's multidimensional model of burnout as the guiding theoretical framework [6]. This model is particularly relevant as it incorporates the interaction between personal experiences, institutional culture, and emotional coping mechanisms—an approach that aligns well with the ethnographic realities of paediatric oncology practice [2].

Compared to adult oncologists, paediatric oncologists experience more intense and prolonged emotional responses, particularly due to the perceived tragedy of a child's death [14]. The ongoing emotional investment in the lives of young patients and their families frequently tests the psychological resilience of clinicians [14]. However, emotional exhaustion is not solely the result of grief over patient loss. Systemic issues—such as excessive workload, insufficient staffing, and inefficient institutional processes—also significantly contribute to burnout [15].

Maslach's model posits that burnout is not merely a byproduct of emotionally taxing work, but rather the result of chronic mismatches between workplace demands and personal capacities, including inadequate support, lack of autonomy, and value conflict [6]. Our findings support this perspective.

Depersonalisation was frequently identified as a coping mechanism—used by clinicians to shield themselves from recurring grief—but over time, this strategy may become maladaptive [14]. It risks eroding the therapeutic alliance with patients and families and may contribute to professional disengagement and moral distress [10]. Poor communication within teams and inter‐professional conflicts exacerbate this issue, undermining emotional cohesion and reducing clinicians' ability to provide empathetic care [16].

The third core dimension of burnout—reduced personal accomplishment—was also evident. Many oncologists reported a diminished sense of efficacy due to bureaucratic overload, procedural rigidity, and limited resources [2]. Negative feedback from bereaved families and poor support during medical training further amplified feelings of ineffectiveness and dissatisfaction [14]. In this light, burnout should be understood not just as an individual failing but as a reflection of systemic dysfunction [2, 14].

Furthermore, paediatric oncologists often operate in ethically complex environments, especially in end‐of‐life care. The review reinforces that moral distress frequently arises not from ethical ambiguity, but from structural barriers that prevent clinicians from acting in accordance with their values [10]. This disconnect fosters emotional frustration, disrupts team dynamics, impairs decision‐making, and contributes to workforce attrition [21].

Despite these challenges, the review highlights numerous coping strategies employed by clinicians. Time with family, exercise, emotional reflection, and peer support serve as important buffers against burnout [2, 9]. The protective value of multidisciplinary teams and shared emotional processing was repeatedly noted [9]. However, the stigma surrounding help‐seeking remains a barrier, especially among trainees—suggesting the need for confidential, accessible mental health resources and emotionally intelligent leadership [11].

Although burnout, emotional exhaustion, and moral distress are well‐recognised challenges in paediatric oncology, this review offers a distinct contribution by consolidating fragmented evidence and highlighting gaps not previously examined [18, 19]. Our findings show that burnout is a multi‐layered phenomenon shaped not only by the emotional demands of paediatric cancer care but also by organisational culture and systemic inefficiencies [2]. The review identifies key gaps, including limited research from low‐ and middle‐income countries, the lack of standardized tools to assess clinician wellbeing, and wide variation in coping strategies and institutional supports [18, 19, 20]. By mapping these patterns, the review highlights the importance of context‐specific mental health interventions, enhanced organizational support systems, and more rigorous research to evaluate effective strategies for enhancing clinician well‐being [20].

### 4.1 Strengths and Limitations

This review synthesizes evidence from both qualitative and quantitative studies, providing a comprehensive and multidimensional perspective on the emotional, moral, and organizational challenges faced by paediatric oncologists. Including studies from diverse healthcare settings and geographic regions enhanced the analysis by capturing varying institutional cultures and resilience frameworks. A key strength lies in the use of Maslach and Leiter's multidimensional model, which provides a robust theoretical foundation for interpreting burnout in a conceptually grounded way.

In addition to identifying risk factors, this review also highlights coping mechanisms, peer support systems, and resilience strategies—offering a more balanced and solution‐focused perspective rather than concentrating solely on deficits or vulnerabilities.

However, some limitations must be acknowledged. Variability in study design, methodology, and outcome measures made it challenging to directly compare findings across studies. Most studies were cross‐sectional, providing limited insight into the long‐term evolution of burnout and resilience. The lack of longitudinal data restricts our ability to assess how psychological challenges progress over time or how interventions may influence outcomes.

Furthermore, most studies were conducted in high‐income countries, with limited representation from low‐ and middle‐income regions. This limits the generalisability of findings to resource‐constrained settings. The focus on paediatric oncologists—while intentional—excluded other multidisciplinary team members such as nurses and social workers, whose experiences might further enrich the understanding of emotional burden in paediatric oncology.

Lastly, potential publication bias remains a concern, as studies reporting significant results are more likely to be published and included in reviews.

### 4.2 Clinical Implications

This scoping review highlights the urgent need for both systemic and individual‐level interventions to address burnout among paediatric oncologists. The findings reveal that emotional exhaustion, moral distress, and depersonalisation are prevalent and significantly impact clinicians' well‐being, job satisfaction, and patient care quality. Clinically, these issues advocate for the integration of trauma‐informed leadership, confidential mental health services, and structured debriefing sessions within paediatric oncology units. Institutions should prioritise resilience‐building programs, provide protected time for emotional recovery, and peer support mechanisms to foster a psychologically safe work environment. Moreover, the stigma surrounding help‐seeking, particularly among trainees, necessitates culturally sensitive and confidential support systems. Addressing organisational contributors—such as excessive workloads, role ambiguity, and bureaucratic constraints—can enhance clinician retention and improve the therapeutic alliance with patients and families. Ultimately, recognising burnout as a multidimensional phenomenon shaped by personal, ethical, and institutional factors is essential for designing targeted interventions that promote clinician well‐being and sustain compassionate paediatric cancer care.

## 5 Conclusions

This scoping review highlights the profound psychological burden experienced by paediatric oncologists, including burnout, emotional exhaustion, and moral distress. These challenges are intensified by high workloads, role ambiguity, inadequate staffing, and limited institutional support.

While personal coping strategies such as peer support, exercise, and emotional detachment help mitigate some of this distress, they are not sufficient on their own. Organisational interventions—such as structured mental health services, workload redistribution, bereavement debriefings, and emotionally supportive leadership—are essential for promoting long‐term clinician well‐being and job satisfaction.

Healthcare systems and policymakers must recognise burnout as both a personal and systemic issue and invest in evidence‐informed interventions that protect clinicians' mental health. Creating supportive work environments is crucial to sustaining the emotional resilience of paediatric oncologists and maintaining high standards of compassionate care.

Future research should evaluate the long‐term impact of these interventions and explore the lived experiences of paediatric oncologists across different cultural and resource settings. Institutions should prioritise resilience training programs and ensure confidential, stigma‐free access to psychological support services as the immediate next steps.

## Author Contributions

**Harshitha D:** project administration, investigation, conceptualisation, resources, writing – original draft. **Vani Verma:** investigation, writing – review and editing. **Arun Ghoshal:** conceptualisation, writing – review and editing. **Divya Sussana Patil:** data curation, writing – review and editing. **Krithika S. Rao:** supervision, writing – review and editing. **Seema R. Rao:** writing – review and editing. **Amrtavarshini R:** writing – review and editing. **Vasudeva Bhat K:** project administration, supervision, conceptualisation, writing – review and editing. **Naveen Salins:** conceptualisation, supervision, writing – review and editing.

## Funding

H.D., and V.V. are funded by the Indian Council of Medical Research [ICMR], a government agency. Grant No. 5/13/18/NS/ICRC/2022/NCD‐III; E‐Office ID:135492.

## Ethics Statement

This scoping review was exempt from ethical approval as it involved the analysis of published literature.

## Conflicts of Interest

The authors declare no conflicts of interest.

## Supporting information


Supporting Information S1

Supporting Information S2
