# Supplementary material for: Burnout and Coping Strategies Among Paediatric Oncologists: A Scoping Review
Source: Psychooncology. 2026 Jan 5;35(1):e70359. doi: 10.1002/pon.70359 (PMC12767751; doi:10.1002/pon.70359)
Supplement: Supplementary file 1 [file PON-35-e70359-s001.odt]

Supplementary file 2

PRISMA-ScR Checklist

| Section | Item | Checklist Item | Reported in Manuscript |
| --- | --- | --- | --- |
| TITLE | 1 | Identify the report as a scoping review. | Title: Yes |
| ABSTRACT | 2 | Provide a structured summary including background, objectives, eligibility criteria, sources of evidence, charting methods, results, and conclusions. | Yes |
| INTRODUCTION | 3 | Describe the rationale for the review in the context of what is already known. | Yes |
|  | 4 | Provide an explicit statement of the questions and objectives being addressed. | Yes (under “Review Question”) |
| METHODS | 5 | Indicate whether a review protocol exists, if and where it can be accessed. | Yes (OSF link provided) |
|  | 6 | Specify eligibility criteria. | Yes |
|  | 7 | Describe all information sources in the search. | Yes |
|  | 8 | Present the full electronic search strategy for at least one database. | Yes (Supplementary File 1) |
|  | 9 | State the process for selecting sources of evidence. | Yes |
|  | 10 | Describe the data charting process. | Yes (described under thematic analysis and data synthesis) |
|  | 11 | List and define all variables for which data were sought. | Yes (burnout, emotional distress, coping strategies, etc.) |
|  | 12 | If critical appraisal was conducted, describe methods and rationale. | Not applicable (scoping reviews typically do not include critical appraisal) |
|  | 13 | Describe methods of handling and summarising the data. | Yes (thematic synthesis described) |
| RESULTS | 14 | Provide number of sources screened, assessed for eligibility, and included, with reasons for exclusions. | Yes (described in PRISMA flow chart) |
|  | 15 | Present characteristics of included sources of evidence. | Yes (Table 1 summarized) |
|  | 16 | Present relevant results that relate to the review questions and objectives. | Yes |
|  | 17 | If critical appraisal was conducted, present data. | Not applicable |
|  | 18 | Summarise and/or present charting results. | Yes (thematic domains presented) |
| DISCUSSION | 19 | Summarise main results, link to review questions and objectives, and consider relevance. | Yes |
|  | 20 | Discuss limitations of the scoping review process. | Yes |
|  | 21 | Provide general interpretation of results and implications for future research, practice, and policy. | Yes |
| FUNDING | 22 | Describe sources of funding for the included sources of evidence and for the scoping review. | Yes (ICMR funding acknowledged) |
