# Supplementary material for: Burnout and Coping Strategies Among Paediatric Oncologists: A Scoping Review
Source: Psychooncology. 2026 Jan 5;35(1):e70359. doi: 10.1002/pon.70359 (PMC12767751; doi:10.1002/pon.70359)
Supplement: Supplementary file 2 [file PON-35-e70359-s002.odt]

Supplementary file 1: Detailed literature search strategy

| PubMed |
| --- |
| Sl. No | Search strategy | Results | Date |
| #1 | "pediatric oncology providers"[Title/Abstract] OR "pediatric oncologists"[Title/Abstract] OR "pediatric oncology"[Title/Abstract] OR "pediatric oncology physicians"[Title/Abstract] OR "pediatric oncology experts"[Title/Abstract] OR "Pediatric oncology professionals"[Title/Abstract] OR "Pediatric hematologist-oncologists"[Title/Abstract] OR "Pediatric oncology staff"[Title/Abstract] OR "oncologists"[MeSH Terms] OR "oncologist"[Text Word] OR "physicians"[MeSH Terms] OR "physicians"[Text Word] | 543465 | 25/05/2025 |
| #2 | "psychological experience*"[Title/Abstract] OR "mental health"[MeSH Terms] OR "mental health"[Text Word] OR "Psychological Well-Being"[MeSH Terms] OR "emotional well being"[Title/Abstract] OR "Psychological Well-Being"[Title/Abstract] OR "burnout, professional"[MeSH Terms] OR "professional burnout"[Text Word] OR "Subjective Stress"[MeSH Terms] OR "Subjective Stress"[Title/Abstract] OR "stress, psychological"[MeSH Terms] OR "psychological stress"[Title/Abstract] OR "coping skills"[MeSH Terms] OR "coping skill*"[Title/Abstract] OR "resilience, psychological"[MeSH Terms] OR "psychological resilience"[Text Word] OR "compassion fatigue"[MeSH Terms] OR "compassion fatigue"[Text Word] OR "job satisfaction"[MeSH Terms] OR "job satisfaction"[Text Word] OR "psychiatric morbidity"[Text Word] OR "emotional exhaustion"[MeSH Terms] OR "emotional exhaustion*"[Text Word] OR "anxiety"[MeSH Terms] OR "anxiety"[Text Word] | 821001 | 25/05/2025 |
| #3 | "pediatric cancer centers"[Text Word] OR "Pediatric palliative care"[Text Word] OR "paediatric oncology settings"[Text Word] OR "pediatric oncology settings"[Text Word] OR "oncology service, hospital"[MeSH Terms] OR "hospital oncology service"[Text Word] OR "oncology setting"[Title/Abstract] OR "cancer care facilities"[MeSH Terms] OR "cancer care facility"[Text Word] OR "cancer care unit"[Text Word] OR "Pediatric oncology units"[Title/Abstract] OR "hospitals, pediatric"[MeSH Terms] OR "pediatric hospital"[Text Word] | 30689 | 25/05/2025 |
|  | ("pediatric oncology providers"[Title/Abstract] OR "pediatric oncologists"[Title/Abstract] OR "pediatric oncology"[Title/Abstract] OR "pediatric oncology physicians"[Title/Abstract] OR "pediatric oncology experts"[Title/Abstract] OR "Pediatric oncology professionals"[Title/Abstract] OR "Pediatric hematologist-oncologists"[Title/Abstract] OR "Pediatric oncology staff"[Title/Abstract] OR "oncologists"[MeSH Terms] OR "oncologist"[Text Word] OR ("physicians"[MeSH Terms] OR "physicians"[Text Word])) AND ("psychological experience*"[Title/Abstract] OR "mental health"[MeSH Terms] OR "mental health"[Text Word] OR "Psychological Well-Being"[MeSH Terms] OR "emotional well being"[Title/Abstract] OR "Psychological Well-Being"[Title/Abstract] OR "burnout, professional"[MeSH Terms] OR "professional burnout"[Text Word] OR "Subjective Stress"[MeSH Terms] OR "Subjective Stress"[Title/Abstract] OR "stress, psychological"[MeSH Terms] OR "psychological stress"[Title/Abstract] OR "coping skills"[MeSH Terms] OR "coping skill*"[Title/Abstract] OR "resilience, psychological"[MeSH Terms] OR "psychological resilience"[Text Word] OR "compassion fatigue"[MeSH Terms] OR "compassion fatigue"[Text Word] OR "job satisfaction"[MeSH Terms] OR "job satisfaction"[Text Word] OR "psychiatric morbidity"[Text Word] OR "emotional exhaustion"[MeSH Terms] OR "emotional exhaustion*"[Text Word] OR ("anxiety"[MeSH Terms] OR "anxiety"[Text Word])) AND ("pediatric cancer centers"[Text Word] OR "Pediatric palliative care"[Text Word] OR "paediatric oncology settings"[Text Word] OR "pediatric oncology settings"[Text Word] OR "oncology service, hospital"[MeSH Terms] OR "hospital oncology service"[Text Word] OR "oncology setting"[Title/Abstract] OR ("cancer care facilities"[MeSH Terms] OR "cancer care facility"[Text Word]) OR "cancer care unit"[Text Word] OR "Pediatric oncology units"[Title/Abstract] OR ("hospitals, pediatric"[MeSH Terms] OR "pediatric hospital"[Text Word])) | 195 | 25/05/2025 |
| Proquest | 41 | 25/05/2025 |
|  | (TI,AB("pediatric oncology providers") OR TI,AB("pediatric oncologists") OR TI,AB("pediatric oncology") OR TI,AB("pediatric oncology physicians") OR TI,AB("pediatric oncology experts") OR TI,AB("Pediatric oncology professionals") OR TI,AB("Pediatric hematologist-oncologists") OR TI,AB("Pediatric oncology staff") OR TI,AB,IF(oncologist) OR TI,AB,IF(physicians)) AND (TI,AB("psychological experience*") OR TI,AB,IF("mental health") OR TI,AB("emotional well being") OR TI,AB("Psychological Well-Being") OR TI,AB,IF("professional burnout") OR TI,AB("Subjective Stress") OR TI,AB("psychological stress") OR TI,AB("coping skill*") OR OR TI,AB,IF("psychological resilience") OR TI,AB,IF("compassion fatigue") OR TI,AB,IF("job satisfaction") OR TI,AB,IF("psychiatric morbidity") OR TI,AB,IF("emotional exhaustion*") OR TI,AB,IF(anxiety)) AND (TI,AB,IF("pediatric cancer centers") OR TI,AB,IF("Pediatric palliative care") OR TI,AB,IF("paediatric oncology settings") OR TI,AB,IF("pediatric oncology settings") OR TI,AB,IF("hospital oncology service") OR TI,AB("oncology setting") OR TI,AB,IF("cancer care facility") OR TI,AB,IF("cancer care unit") OR TI,AB("Pediatric oncology units") OR TI,AB,IF("pediatric hospital")) |  |  |
| SCOPUS |  | 465 | 25/05/2025 |
|  | ( TITLE-ABS ( "pediatric oncology providers" ) OR TITLE-ABS ( "pediatric oncologists" ) OR TITLE-ABS ( "pediatric oncology" ) OR TITLE-ABS ( "pediatric oncology physicians" ) OR TITLE-ABS ( "pediatric oncology experts" ) OR TITLE-ABS ( "Pediatric oncology professionals" ) OR TITLE-ABS ( "Pediatric hematologist-oncologists" ) OR TITLE-ABS ( "Pediatric oncology staff" ) OR INDEXTERMS ( oncologists ) OR TITLE-ABS-KEY ( oncologist ) OR INDEXTERMS ( physicians ) OR TITLE-ABS-KEY ( physicians ) ) AND ( TITLE-ABS ( "psychological experience*" ) OR INDEXTERMS ( "mental health" ) OR TITLE-ABS-KEY ( "mental health" ) OR INDEXTERMS ( "Psychological Well-Being" ) OR TITLE-ABS ( "emotional well being" ) OR TITLE-ABS ( "Psychological Well-Being" ) OR INDEXTERMS ( "burnout, professional" ) OR TITLE-ABS-KEY ( "professional burnout" ) OR INDEXTERMS ( "Subjective Stress" ) OR TITLE-ABS ( "Subjective Stress" ) OR INDEXTERMS ( "stress, psychological" ) OR TITLE-ABS ( "psychological stress" ) OR INDEXTERMS ( "coping skills" ) OR TITLE-ABS ( "coping skill*" ) OR INDEXTERMS ( "resilience, psychological" ) OR TITLE-ABS-KEY ( "psychological resilience" ) OR INDEXTERMS ( "compassion fatigue" ) OR TITLE-ABS-KEY ( "compassion fatigue" ) OR INDEXTERMS ( "job satisfaction" ) OR TITLE-ABS-KEY ( "job satisfaction" ) OR TITLE-ABS-KEY ( "psychiatric morbidity" ) OR INDEXTERMS ( "emotional exhaustion" ) OR TITLE-ABS-KEY ( "emotional exhaustion*" ) OR INDEXTERMS ( anxiety ) OR TITLE-ABS-KEY ( anxiety ) ) AND ( TITLE-ABS-KEY ( "pediatric cancer centers" ) OR TITLE-ABS-KEY ( "Pediatric palliative care" ) OR TITLE-ABS-KEY ( "paediatric oncology settings" ) OR TITLE-ABS-KEY ( "pediatric oncology settings" ) OR INDEXTERMS ( "oncology service, hospital" ) OR TITLE-ABS-KEY ( "hospital oncology service" ) OR TITLE-ABS ( "oncology setting" ) OR INDEXTERMS ( "cancer care facilities" ) OR TITLE-ABS-KEY ( "cancer care facility" ) OR TITLE-ABS-KEY ( "cancer care unit" ) OR TITLE-ABS ( "Pediatric oncology units" ) OR INDEXTERMS ( "hospitals, pediatric" ) OR TITLE-ABS-KEY ( "pediatric hospital" ) ) |  |  |
| Web of Science | 117 | 25/05/2025 |
| #1 | ALL=("pediatric oncology providers" OR "pediatric oncologists" OR "pediatric oncology" OR "pediatric oncology physicians" OR "pediatric oncology experts" OR "Pediatric oncology professionals" OR "Pediatric hematologist-oncologists" OR "Pediatric oncology staff" OR oncologists OR oncologist OR physicians OR physicians) | 5369204 |  |
| #2 | ALL=("psychological experience*" OR "mental health" OR "mental health" OR "Psychological Well-Being" OR "emotional well being" OR "Psychological Well-Being" OR "burnout, professional" OR "professional burnout" OR "Subjective Stress" OR "Subjective Stress" OR "stress, psychological" OR "psychological stress" OR "coping skills" OR "coping skill*" OR "resilience, psychological" OR "psychological resilience" OR "compassion fatigue" OR "compassion fatigue" OR "job satisfaction" OR "job satisfaction" OR "psychiatric morbidity" OR "emotional exhaustion" OR "emotional exhaustion*" OR anxiety OR anxiety) | 1089277 |  |
| #3 | ALL=("pediatric cancer centers" OR "Pediatric palliative care" OR "paediatric oncology settings" OR "pediatric oncology settings" OR "oncology service, hospital" OR "hospital oncology service" OR "oncology setting" OR "cancer care facilities" OR "cancer care facility" OR "cancer care unit" OR "Pediatric oncology units" OR "hospitals, pediatric" OR "pediatric hospital") | 10491 |  |
| #4 | #1 AND #2 AND #3 | 117 |  |
| CINAHL Ultimate | 252 | 25/05/2025 |
|  | ( (TI "pediatric oncology providers" OR AB "pediatric oncology providers") OR (TI "pediatric oncologists" OR AB "pediatric oncologists") OR (TI "pediatric oncology" OR AB "pediatric oncology") OR (TI "pediatric oncology physicians" OR AB "pediatric oncology physicians") OR (TI "pediatric oncology experts" OR AB "pediatric oncology experts") OR (TI "Pediatric oncology professionals" OR AB "Pediatric oncology professionals") OR (TI "Pediatric hematologist-oncologists" OR AB "Pediatric hematologist-oncologists") OR (TI "Pediatric oncology staff" OR AB "Pediatric oncology staff") OR (MH oncologists+) OR oncologist OR (MH physicians+) OR physicians ) AND ( (TI "psychological experience*" OR AB "psychological experience*") OR (MH "mental health+") OR "mental health" OR (MH "Psychological Well-Being+") OR (TI "emotional well being" OR AB "emotional well being") OR (TI "Psychological Well-Being" OR AB "Psychological Well-Being") OR (MH "burnout, professional+") OR "professional burnout" OR (MH "Subjective Stress+") OR (TI "Subjective Stress" OR AB "Subjective Stress") OR (MH "stress, psychological+") OR (TI "psychological stress" OR AB "psychological stress") OR (MH "coping skills+") OR (TI "coping skill*" OR AB "coping skill*") OR (MH "resilience, psychological+") OR "psychological resilience" OR (MH "compassion fatigue+") OR "compassion fatigue" OR (MH "job satisfaction+") OR "job satisfaction" OR "psychiatric morbidity" OR (MH "emotional exhaustion+") OR "emotional exhaustion*" OR (MH anxiety+) OR anxiety ) AND ( "pediatric cancer centers" OR "Pediatric palliative care" OR "paediatric oncology settings" OR "pediatric oncology settings" OR (MH "oncology service, hospital+") OR "hospital oncology service" OR (TI "oncology setting" OR AB "oncology setting") OR (MH "cancer care facilities+") OR "cancer care facility" OR "cancer care unit" OR (TI "Pediatric oncology units" OR AB "Pediatric oncology units") OR (MH "hospitals, pediatric+") OR "pediatric hospital" ) |  |  |
